# The Safety of Novel Therapies in Chronic Lymphocytic Leukemia in the Era of Intermittent Fasting: A Pharmacology-Based Review

**DOI:** 10.3390/cancers16112079

**Published:** 2024-05-30

**Authors:** Maria Benkhadra, Nuha Fituri, Soha Aboukhalaf, Rola Ghasoub, Mervat Mattar, Khalil Alfarsi, Salem Alshemmari, Mohamed A. Yassin

**Affiliations:** 1 Department of Pharmacy, National Center for Cancer Care and Research, Hamad Medical Corporation, Doha P.O. Box 3050, Qatar; rghasoub@hamad.qa; 2 College of Medicine, QU Health, Qatar University, Doha P.O. Box 2713, Qatar; nfituri@hamad.qa (N.F.); saboukhalaf@hamad.qa (S.A.); 3 Clinical Hematology Unit, Internal Medicine Department, Kasr Al Ainy Faculty of Medicine, Cairo University, Cairo 12111, Egypt; mermattar@kasralainy.edu.eg; 4 Department of Hematology, Sultan Qaboos University Hospital Muscat, Seeb P.O. Box 35, Oman; khalilf@squ.edu.om; 5 Department of Medicine, Faculty of Medicine and Department of Hematology, Kuwait Cancer Control Centre, Shuwaikh P.O. Box 42262, Kuwait; salem.alshammari@ku.edu.kw; 6 Department of BMT/Hematology, National Center for Cancer Care and Research, Hamad Medical Corporation, Doha P.O. Box 3050, Qatar

**Keywords:** chronic lymphocytic leukemia, tumor lysis syndrome, gastrointestinal bleeding, intermittent fasting, fluid-restricted intermittent fasting, fluid-liberal intermittent fasting, Bruton tyrosine kinase inhibitors (BTK), BCL2 inhibitors

## Abstract

**Simple Summary:**

The recent growing interest in intermittent fasting (IF) is attributed to its preclinical benefits in various aspects of cancer management. Novel agents, namely, venetoclax and Bruton tyrosine kinase inhibitors (BTKIs), are widely used in the frontline setting of chronic lymphocytic leukemia (CLL). However, tumor lysis syndrome (TLS) and gastrointestinal bleeding (GIB) are particularly concerning for patients. This narrative review aimed to pioneer the exploration of the potential implications of IF for CLL patients receiving novel agents, focusing on GIB and TLS risks. In fluid-restricted IF, there is a higher risk of TLS with venetoclax due to dehydration, while in fluid-liberal IF, its absorption can be reduced. Moreover, during fasting conditions, the levels of gastric acid increase along with the risk of GIB in patients receiving BTKis. In the context of IF in CLL, further research is warranted to establish the safety of IF in CLL patients on novel agents.

**Abstract:**

Intermittent fasting (IF) has recently gained popularity due to its emerging benefits in reducing weight and improving metabolic health. Concurrently, novel agents (NAs) like venetoclax and Bruton tyrosine kinase inhibitors (BTKIs) have revolutionized the treatment of chronic lymphocytic leukemia (CLL). Unfortunately, it is unclear whether the associated risks of tumor lysis syndrome (TLS) and gastrointestinal bleeding (GIB) are increased in IF practitioners receiving NAs. This review explored the literature available on the permissibility of IF in CLL patients undergoing treatment with first-line NAs (FLNAs). Literature was scoped to identify IF patterns and the available data on TLS and GIB risks associated with food and fluid intake in CLL patients receiving FLNAs. Although current evidence is insufficient to recommend IF in this population, it may be possible for patients on venetoclax to conservatively practice fluid-liberal IF, provided that adequate hydration and the consistent administration of food are achieved. In contrast, considering the significant risk of TLS and the pharmacokinetics of venetoclax, patients should be discouraged from practicing fluid-restricted IF, especially during the ramp-up phase. Moreover, patients on BTKIs ought to refrain from IF due to the possible risk of GIB until further data are available. Further research is needed to provide conclusive recommendations.

## 1. Introduction

Chronic lymphocytic leukemia (CLL) is a neoplasm that affects mature B cells and is characterized by the expansion of the immunologically incompetent monoclonal B lymphocytes [1]. In the United States, CLL accounts for around 25% of new cases of leukemia [2]. Conventionally, CLL was treated with chemoimmunotherapy through fludarabine-based regimens in combination with the anti-CD20 monoclonal antibody rituximab. Recent developments in the treatment of CLL have resulted in the introduction of novel agents (inhibitors of BCL2 and Bruton tyrosine kinase [BTK]). Because of their favorable survival outcomes, these treatments have largely replaced chemoimmunotherapy, whether alone or in combination with other targeted therapies in first-line and relapsed/refractory settings [3,4].

Venetoclax, the only approved BCL-2 inhibitor to date, works by restoring the impaired apoptotic activity of the affected B-cells, thereby reducing uncontrolled cellular proliferation (details in Section 4.1) [5]. By potently reactivating apoptosis, venetoclax causes rapid lysis of the CLL cells; hence, its main concerning side effect remains tumor lysis syndrome (TLS). The incidence of TLS with venetoclax ranged from 2.7% to 8.9% (with fatalities) in phase I clinical trials [6]. Therefore, adequate strategies to prevent TLS, such as gradual dose escalation, hydration, and urate-lowering therapies, are crucial for the safe administration of venetoclax (details in Section 5.1) [6].

Another mechanism by which B-cell proliferation is enhanced in CLL cells is the activation of the B-cell receptor (BCR), regardless of the presence of an antigen (ligand-independent activation) [7]. BCR activation initiates a multistep downstream cascade, including BTK and dependent pathways, resulting ultimately in increased DNA transcription and higher cellular proliferation. BTK inhibitors, such as ibrutinib, acalabrutinib, and zanubrutinib, inhibit BTK and dependent pathways, thereby reducing CLL cell survival [7]. 

Unfortunately, BTK inhibitors have the potential to inhibit other kinases, such as the Tec family of kinases that are important for platelet signaling [8,9]. This leads to an increased risk of bleeding events [8,9]. The incidence of bleeding varies among the different BTK inhibitors due to the variability in their selectivity toward BTK inhibition and the extent of their off-target inhibitions [10]. Major hemorrhage (higher than grade 3), including gastrointestinal bleeding (GIB), was reported in 4%, 3%, and 2% of ibrutinib, acalabrutinib and zanubrutinib patients, respectively, in clinical trials [8].

Despite the presence of risk-mitigating recommendations in the medical literature for the care of patients using venetoclax and BTK inhibitors, TLS and GIB remain active concerns for the care of CLL patients, respectively [8,9,10,11,12,13]. Moreover, in recent years, intermittent fasting (IF), defined as a period of voluntary abstinence from food and/or drink [14], has gained popularity. This is due to its benefits in reducing weight and improving metabolic health, as shown by Varady et al. [15]. Furthermore, preclinical and animal studies have suggested that IF holds potential for improving the response to chemotherapeutic agents and reducing related side effects and cancer proliferation [16]. The mechanism of those possible benefits is hypothesized to be related to the role of IF in weight reduction, the modulation of glucose and insulins levels, and increases in oxidative phosphorylation. This leads to the release of reactive oxygen species (ROS) and the induction of apoptosis in cancer cells [17,18].

In addition, Nista et al. demonstrated that the gut microbiota may contribute to the lack of response to some anticancer therapies in pancreatic cancer [19]. Even in non-cancer conditions, the microbiota is thought to affect the oral bioavailability of orally administered medications [20]. In CML, Yassin et al. illustrated that IF did not significantly affect hematological and BCR::ABL outcomes in patients receiving tyrosine kinase inhibitors [14]. In CLL, a high variability in the gut microbiota was observed among patients, irrespective of the treatment received [21]. However, whether this variability has any effect on clinical disease outcomes or response to therapy is still unclear.

Various types of IF exist (Section 3), with some involving abstinence from a certain or any type of food with or without fluids for a certain duration of time. Some may be part of a nutritional choice that is practiced all year or pertain to religious traditions that are usually shorter in duration. For example, Ramadan fasting runs for 29 to 30 consecutive days per year, during which practitioners abstain from food and water from sunrise to sunset. This may expose them to dehydration [22], which is a risk factor for developing TLS in patients with hematologic malignancies [23]. Furthermore, during this period of fasting, gastric acid levels rise [24], along with the chances of GIB in high-risk patients [25]. Whether this risk increases with the use of BTK inhibitors is still unclear. 

The current knowledge about the effects of IF on the side effects of venetoclax and BTK inhibitors, particularly TLS and GIB, is scarce. Therefore, this review aims to explore the literature available to investigate the permissibility of IF in CLL patients who are being treated with the first-line novel agents (FLNAs) venetoclax or BTK inhibitors. This was to identify the possible effects that fasting conditions may have on the risks of GIB and TLS as well as on the pharmacokinetics of BTK and BCL-2 inhibitors. The ultimate target is to direct future research by shedding light on the current gaps in the literature.

## 2. Methodology

Initially, a literature search was conducted to identify the different types of IF practices to categorize them according to the groups of consumables abstained from (food only or food with beverages). This was followed by another literature exploration of the possible effects of fasting conditions and fluid restriction on the pharmacokinetics (PK) of FLNAs, as reported by PK studies and the FDA labels of the medications. Moreover, pre- and post-marketing reports were scoped for data on TLS and GIB risks associated with FLNAs in CLL patients. Thereafter, the effects of fasting and or fluid-restricted states on GIB and TLS risks were identified. Lastly, all the risks found were accumulated to build a pathway for the permissibility of IF in this patient population based on the current knowledge of the topic.

## 3. Types of Intermittent Fasting

IF patterns differ between religious and non-religious practices primarily in the duration of fasting and the consumables abstained from during fasting periods. For example, during the holy month of Ramadan, conventionally a form of 16:8 fasting, practitioners abstain from food and beverages during fasting hours for 29 to 30 days each year (fluid-restricted IF). However, other non-religious forms of 16:8 fasting may be fluid-liberal, i.e., they allow water and non-caloric beverages during fasting hours [26,27]. As shown in Table 1, religious IF types are limited to specific durations and days per year, while non-religious IF practices are usually nutritional choices that are not limited to certain durations [26,27]. Understanding the type of fasting patients are practicing (Table 1) is vital to evaluate the risk of fasting with CLL treatments (Section 6).

## 4. First-Line Novel Therapies

### 4.1. BTK Inhibitors

**Figure 1:**
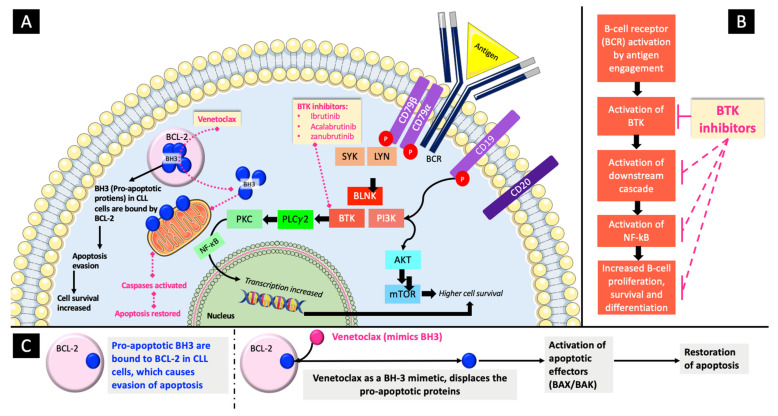
Illustration of the role of BCL-2 and BTK in B-cell malignancies with the mechanisms of action of venetoclax and BTKi. (**A**). Intracellular illustration of the mechanisms of action of venetoclax and BTK inhibitors. (**B**). A closer look at the mechanism of action of BTK inhibitors (the solid line shows direct inhibition while the dotted line shows indirect inhibition because of the inhibition of BTK). (**C**). An in-depth insight into the mechanism of action of venetoclax.

Bruton tyrosine kinases (BTK) are non-receptor kinases that are expressed in many cells, primarily hematopoietic B-cells, myeloid cells, and lymphoid cells. Upon the activation of B-cell receptors (BCR), BTKs are recruited by cell membranes where they are phosphorylated and hence activated. This results in a multistep downstream cascade that results in the activation of NF-kB pathway. This increases DNA transcription and cell proliferation and survival (Figure 1A,B). The currently approved BTK inhibitors for CLL treatment (ibrutinib, acalabrutinib, and zanubrutinib) all bind to the C481 site in BTK, thereby blocking its kinase activity [28,29,30].

### 4.2. BCL-2 Inhibitors

In CLL, the pro-apoptotic BH3-only proteins are bound to BCL2, thereby allowing the leukemic cells to evade apoptosis. Venetoclax is a small and selective oral BCL-2 inhibitor with a high affinity for binding to the BH3′s binding site on BCL2 (Figure 1A,C). This leads to the displacement of the BH3-only proteins, the activation of the apoptotic effectors (BAX and BAK), and the restoration of apoptosis in tumor cells [31].

## 5. TLS in CLL

TLS is a relatively uncommon but potentially life-threatening oncologic and metabolic emergency that is associated with the treatment of cancers, especially hematological malignancies [32]. The fifth version of the Common Terminology Criteria for Adverse Events (CTCAE) has defined TLS as a disorder highlighted by metabolic derangements that are caused by the rapid lysis of tumor cells, whether spontaneously or due to the initiation of therapy [33]. The abrupt death of tumor cells results in the release of their intracellular contents into the bloodstream. This raises the serum concentrations of uric acid, potassium, and phosphate while simultaneously decreasing the serum concentration of calcium [34]. These metabolic derangements can lead to significant morbidities ranging from renal insufficiency, seizures, neurological complications, and cardiac arrythmias to possibly death if not promptly addressed [35]. In the current era of novel therapies and highly effective cytoreductive agents, the incidence of TLS in malignancies that were previously rarely associated with it, such as CLL, is being increasingly reported in the literature [6,36,37].

Although the risk of developing TLS is the greatest amongst patients treated for hematological malignancies, it varies considerably depending on the tumor type and tumor burden, as well as patient- and treatment-related factors [1,23]. Historically, CLL has not been linked to a high incidence of TLS due to its slow proliferation rate [23,38]. However, targeted therapies that cause the rapid destruction of leukemic cells have intensified the risk of TLS in patients with a high tumor burden of CLL (characterized by the involvement of the lymph nodes, spleen, and liver) [3,23,32,34]. In particular, the discovery of venetoclax has been responsible for the recently marked amplification of the TLS risk in CLL [6,23].

### 5.1. TLS in CLL Patients Treated with Novel Agents

It is essential to identify patients at a high risk of developing TLS to quickly prevent the associated morbidity and mortality [39]. An international TLS expert panel released a risk stratification system for TLS based on the type of malignancy and incorporated all risk factors including tumor burden [40]. The provided recommendations for prevention and management vary according to a patient’s risk category (high, intermediate, low) [41]. 

**Figure 2:**
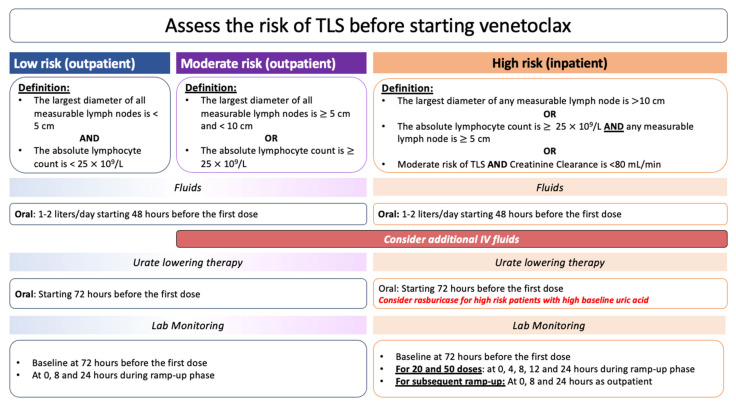
The recommended measures for TLS prevention in CLL patients who are specifically receiving venetoclax.

The use of venetoclax-based therapy has led to the stratification of most CLL patients into intermediate- and high-TLS-risk groups (Figure 2) [38,41]. The literature has warned against TLS incidence with venetoclax therapy due to the associated severity and fatality that was reported in phase I/II studies. This was further emphasized in a systematic review of six phase I studies that investigated the use of venetoclax therapy in diffuse large B-cell lymphoma (DLBCL) and CLL patients. All reported fatalities in those studies had occurred among CLL patients even though, traditionally, CLL was considered to be a lower-risk disease for TLS compared to DLBCL [6]. Therefore, venetoclax initiation is slowly performed over 5 weeks with a gradual increase in the dose (i.e., ramp-up dosing schedule; Table 2) to reduce this risk of TLS.

TLS was rarely reported in the literature as a complication for the treatment of CLL with BTK inhibitors. Few cases were reported among ibrutinib patients including three patients from a phase Ib/II study [36,42]. Even in the landmark clinical trials that compared BTK inhibitors with each other, TLS was reported in less than 1% of the study populations (Table 3) [43,44]. Interestingly, when ibrutinib was used as a debulking strategy (i.e., to reduce the disease burden) for three cycles before the addition of venetoclax (CAPTIVATE phase II trial), there were no TLS cases highlighted within the 27.9-month follow-up [45].

To further formulate an idea of the real-world incidence of TLS risk among CLL patients, the FDA Adverse Events Reporting System (FAERS) was scouted [46]. Records of FAERS implementation until the end of June 2023 were scanned by the database. There was a total of 905 cases of TLS reported among CLL patients in FAERS (those using novel agents, monoclonal antibodies, chemoimmunotherapy, combination therapies, and others). As shown in Table 4, when data were filtered to novel agents as monotherapies, venetoclax had the highest number of cases of TLS reported (*n* = 195), followed by ibrutinib (*n* = 73), acalabrutinib (*n*= 36), and zanubrutinib (*n* = 3). The fatalities reported in the database were also the highest among venetoclax patients (*n* = 68). 

The results inferred from FAERS are insightful but should be interpreted with caution due to multiple limitations. Firstly, it is challenging to correlate each of the events reported with the predisposing patient and disease-related risk factors. For example, it would be important to identify certain factors (e.g., disease stage, tumor burden, concomitant use of monoclonal antibodies, baseline kidney function, and TLS prevention measures) in a case to determine the risk of TLS and whether, as an adverse event, it was indeed caused by the suspected medication. Secondly, the database itself, while rich in adverse event reports, allows for discrepancies in the keywords used to report them, which would yield different search results according to the search terms used. Lastly, the total number of all adverse events reported (unrestricted to TLS) varies drastically among the different agents according to the date of their introduction to the market (Table 3). Hence, it is expected that the newer a medication is, the more likely that the number of adverse events reported on it will be underestimated. Despite these limitations, these data present the currently obtainable real-world data on the incidence of TLS among CLL patients using novel agents other than venetoclax.

### 5.2. TLS Prevention in CLL Patients Treated with Novel Agents

The prompt prevention of TLS is critical in reducing its incidence and fatalities [34]. Adequate hydration is the cornerstone of TLS prophylaxis as it enhances renal perfusion and filtration while minimizing acidosis. This further contributes to the prevention of the deposition of uric acid and calcium phosphate crystals in renal tubules [40]. The 2010 refined and updated International Expert Panel on TLS advises patients in the intermediate- and high-risk groups to receive at least 3 L/m^2^ per day of IV fluid, with close monitoring of urine output to be maintained within 80 to 100 mL/m^2^ per hour [34,47]. The optimally recommended duration of hydration is until the markers of tumor burden have been considerably resolved [13,47,48]. 

TLS prevention among patients treated with BTK inhibitors highly depends on the disease burden, baseline organ function, and tolerability of urate-lowering therapies, rather than the agent itself. As for venetoclax, considering its high TLS risk, specific evidence-based guidance was required for TLS risk mitigation in CLL patients receiving it (Figure 2). This guidance has recommended ramp-up dosing, aggressive oral/intravenous hydration, the use of hypouricemic agents, and the careful monitoring of serum electrolytes, uric acid, and urine output at each dose ramp-up as the main prophylactic strategies [13,36,48]. The guidance also suggests a pathway to recognize patients at a high risk of TLS (high tumor burden and compromised renal function at baseline) to initiate more aggressive prophylactic measures (rasburicase and IV hydration). 

## 6. GIB in CLL

GIB is defined as bleeding that originates at any point within the gastrointestinal tract. It can be categorized either anatomically as upper (UGIB) versus lower GIB (LGIB) or qualitatively as overt or occult [49]. UGIB originates above the ligament of Treitz while LGIB originates below it. Alternatively, overt GIB is defined as that which is symptomatic, acute, and grossly identifiable. On the other hand, occult GIB has a more chronic nature and can only be diagnosed using specialized chemical tests [50]. Peptic ulcer disease (PUD) is the most common cause of UGIB, with around 30–50% of its cases attributed to PUD. Subsequently, esophagitis amounts to 15–20% of all of the UGIB cases [51,52]. In contrast, patients with liver cirrhosis are at the highest risk of variceal bleeds, with almost 50–60% of the cases arising secondary to esophageal varices [53]. 

In addition to PUD and esophagitis, recent research has demonstrated the risk of bleeding diathesis as a complication of acquired von Willebrand disease (VWD) with CLL [50]. Furthermore, it is thought that patients with CLL can develop immune thrombocytopenia, as demonstrated by multiple case reports, which can also increase the risk of bleeding [51,54]. Also, as a result of the progression of direct lymphoproliferation into chronic PUD, patients with CLL can rarely develop GIB regardless of therapy choice [53]. The existence of these comorbidities can pose a risk to patients with CLL. This risk is further exacerbated with the use of BTK inhibitors, which are known to cause bleeding [8]. As such, it is important to consider each patient’s individual risk factors for GIB, particularly when starting novel agents.

BTK inhibitors differ in their inhibitory potency of different enzymes, which explains the intraclass variability in the severity of adverse drug reactions between BTK inhibitors [55]. At the level of BTK, ibrutinib and zanubrutinib had the lowest inhibitory concentrations (IC_50_) and thereby the highest potencies [55]. However, ibrutinib maintained this high potency across other enzymes and receptors, such as TEC, EGFR, ITK, BLK, JAK3, ERBB2, and ERBB4. This off-target activity, particularly at TEC tyrosine kinases, may explain the higher rates of bleeding adverse events with ibrutinib compared to other BTK inhibitors, as illustrated in Table 5 [43,44,55,56]. 

The limitation of solely inspecting the data from the landmark trials is that they do not provide specific information on GIB. Rather, these trials report the cumulative incidence of all bleeding events. Moreover, they do not provide accurate subcategorization of patients who were concomitantly treated with a BTK inhibitor and anticoagulants or antiplatelets. Hence, FAERS was explored again for the reported cases of GIB in CLL patients. There was a total of 377 cases of GIB and CLL reported in the database. The highest number of GIB adverse events was reported among ibrutinib patients (*n* = 191). This was slightly higher among patients who were concomitantly using an anticoagulant or an antiplatelet (*n* = 207), as shown in Table 6 [46]. Given the shortcomings of using FAERS, as highlighted in Section 4.1, there is a need for the systematic collection of real-world evidence on the risk of GIB in CLL patients treated with novel agents. This is of particular importance for the identification of risk factors for GIB in this patient population and possible protective measures.

## 7. Challenges for Fasting in CLL Patients Treated with Novel Agents

### 7.1. The Need for Aggressive Hydration in Fluid-Restricting Fasting Practices

Aggressive hydration is required to prevent the life-threatening manifestations of TLS, particularly in patients treated with venetoclax. This poses an obstacle to patients wishing to practice fluid-restricted IF as it can interfere with the recommended hydration goal [38]. Furthermore, the estimated kidney function plays an integral role in determining the adaptive capacity of patients to combat the metabolic consequences of therapy-induced cell lysis [34]. Therefore, the pre-existing states of low urinary flow caused by dehydration, hypotension, or inadequate hydration during cytoreductive therapy can weaken the kidney’s ability to adjust to the metabolic derangements seen in tumor lysis [35,57]. This increases the risk of developing TLS and intensifies the risk of its detrimental complications [35]. The vitality of adequate hydration for TLS prevention, prior to and during therapy initiation, can hinder patients from practicing fluid-restricted IF by increasing their risk of dehydration.

### 7.2. The Risks of GIB with BTK Inhibitors and Fasting

Studies have indicated that gastric acid levels increase during the holy month of Ramadan, which is a form of 16:8 fluid-restricted fasting [24]. Consequently, a higher incidence of bleeding and PUD complications has been observed during this period of fasting [25]. In contrast, a study conducted on patients with chronic liver disease showed that Ramadan fasting decreased the incidence of variceal bleeding (1%) compared to the non-fasting group (9.1%) [15,58,59]. This suggests that, overall, fasting may carry an increased risk of acute bleeding events, especially in patients with a history of PUD and esophagitis [25].

### 7.3. Possible Interactions with Food 

The phase I and II studies that explored the pharmacokinetics of venetoclax concluded that the exposure of venetoclax and time to peak concentration (C_max_) are affected by fed and fasted states [60]. In fasting patients, the time to C_max_ is delayed by about 2 h, while the exposure (as measured by AUC) is increased by 3 to 5 times when the dose is preceded by food, particularly if high in fat content [61]. This explains the recommendation by drug labels to administer venetoclax with meals.

However, among BTK inhibitors, the data from zanubrutinib’s pharmacokinetic studies have shown that it is the least affected by food intake, as shown in Table 7 [62,63]. As for ibrutinib, its AUC and ability to reach its C_max_ are increased by food intake [64]. Despite these pharmacokinetic properties, ibrutinib’s drug label recommends its administration regardless of food intake. This is based on the assumption that repeated fasting conditions are unlikely [64,65]. Hence, data are scarce in relation to the effects of consecutive fasting for a prolonged period on ibrutinib’s pharmacokinetics. As for acalabrutinib, it exists in tablet and capsule formulations [66,67]. The administration of acalabrutinib after high-fat/high-caloric meals, in either formulation, reduces its C_max_ [66,67]. However, this reduction in C_max_ did not appear to have any significant effect on acalabrutinib’s AUC [66,67]. Therefore, acalabrutinib tablet and capsule drug labels also recommend administration, irrespective of food intake [66,67].

### 7.4. Fasting Practices for a Day or More

Some IF patterns require the practitioner’s food abstinence for 24 h on alternate days (eat-stop-eat approach, as explained in Table 1). Considering that all novel agents require at least daily administration (twice daily in the case of acalabrutinib), this can present a challenge congruent to missing doses of therapy. This can pose a threat to treatment efficacy, especially when consistently practiced. Therefore, it is vital to advise patients against the types of fasting that involve food abstinence for 24 h.

## 8. Pathway for Decision of Fasting

**Figure 3:**
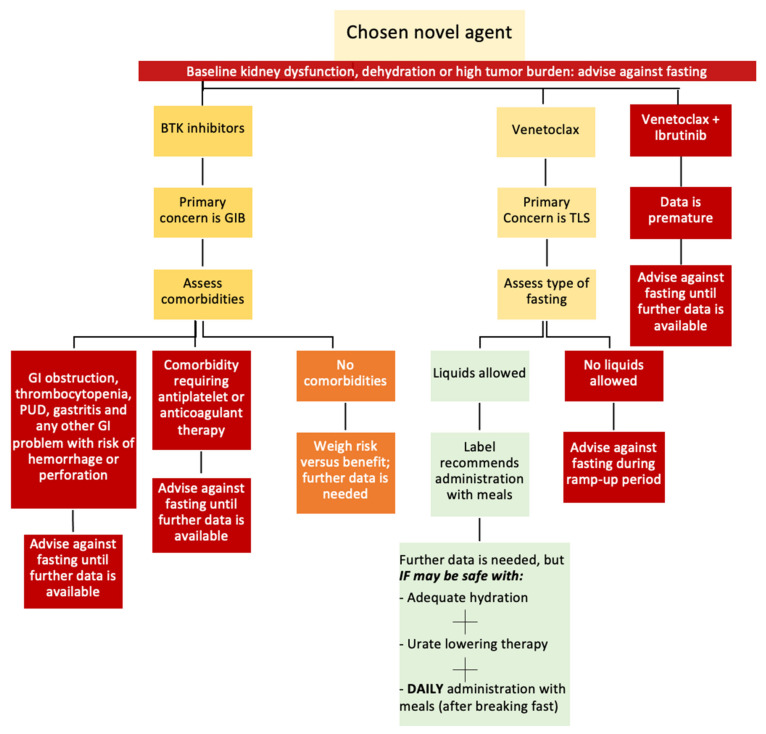
Suggested pathway for IF decision making with first-line novel agents in CLL (Red-colored boxes: fasting is not advised, yellow-colored boxes: primary concern and assessment needed, orange-colored boxes: caution is advised, green-color boxes: conditional fasting may be practiced).

The current evidence is insufficient to recommend IF in CLL patients. However, for patients strongly inclined toward practicing IF, there are major points that we took into consideration for creating the pathway demonstrated in Figure 3. This was to highlight the possible safety issues that may be associated with practicing IF among CLL patients treated with novel agents. These points include the following crucial concerns for each class of novel agent:

- **Venetoclax and TLS**: Attaining adequate hydration is a necessity that may challenge fluid-restricted fasting during the venetoclax ramp-up phase.
- **BTK inhibitors and GIB**: Identifying the risk factors for GIB for each patient is vital. In addition, the increased gastric acidity during fasting periods (fluid-restricted and fluid-liberal IF) can increase the risk of GIB.
- The fasting patterns practiced by CLL patients should accommodate the daily administration of iburinib, zanubrutinib, and venetoclax (after food) and the twice-daily administration of acalabrutinib.
- Comorbidities that can increase the risks for GIB and TLS should be considered before permitting CLL patients to fast.

## 9. Discussion and Future Directions

The role of IF in cancer therapy is under the research spotlight for the potential optimization of cancer therapy outcomes. In a clinical trial described by Groot et al., 13 patients with HER-2 negative breast cancer undergoing neo-adjuvant intravenous (IV) chemotherapy were randomized to fasting versus non-fasting groups [68]. The fasting period for the first group lasted for 24 h before and after chemotherapy days and involved abstinence from food only (fluid-liberal fasting) [68]. The fasting group demonstrated reductions in chemotherapy-related side effects and improved treatment tolerability [68]. Despite the importance and relevance of this topic, limited data are published on the effects of fasting on cancer patients, with most of these trials being qualitative [68,69,70].

In a small case series by Safdie et al., 10 patients who were diagnosed with solid malignancies and underwent IV chemotherapy with fasting reported a reduction in chemotherapy fatigue and weakness while fasting [69]. All of the 10 cases abstained from food for 48–140 hours prior to and/or 5–56 h following IV chemotherapy (fluid-liberal IF) [69]. None of these patients reported significant side effects [69]. 

Moreover, Dorff et al. investigated 20 solid cancer patients who were receiving IV chemotherapy containing platinum-derived agents with multiple IF durations (24, 48, and 72 h) in a dose-escalation and feasibility study [70]. IF involved abstaining from food (fluid-liberal IF) and was found to be safe and applicable for cancer patients [70]. It also demonstrated some preliminary results of lower DNA damage, as shown in the post-chemotherapy leukocyte counts in patients who fasted for 72 h [70].

In line with the previously published data, a comparable safety profile for IF during chemotherapy was observed by Badar et al. [71]. In Saudi Arabia, this clinical trial assessed the safety of chemotherapy administration during IF in Ramadan [71]. The fluid-restricted IF in this study involved daily abstinence from food and beverages from sunrise to sunset for 2 weeks (the study took place in the last 2 weeks of Ramadan) [71]. IV chemotherapy was administered after fasting was broken at sunset [71]. The reporting of adverse events was obtained daily and compared between the 2 weeks of fasting and another 2 weeks after Ramadan during which no fasting took place [71]. The chemotherapy regimen was remained the same throughout the 4 weeks of the study [71]. Eleven cancer patients (four hematological malignancies and seven solid tumors cases) were followed [71]. Badar T et al. concluded that chemotherapy administration during IF in the month of Ramadan was safe [71]. On the other hand, a systematic review and meta-analysis (SR/MA) by Ferro et al. showed that there is no significant amelioration in chemotherapy-related adverse events amongst IF patients [72]. All the studies included in this SR/MA were on solid cancer patients who underwent IV chemotherapy [72]. 

Hence, the results presented here attempt to fill many of the current gaps in the existing literature. Firstly, the existing literature focuses on solid cancer patients who are rarely at risk for TLS compared to hematological malignancy patients [73]. Therefore, the possible risks associated with fluid-restricted IF are more relevant to hematology patients receiving potent therapies. In addition to that, the anticancer medications covered in the existing literature are all IV chemotherapies, while, in this review, we present orally administered targeted agents that rely on multiple factors for their bioavailability. These factors could include administration with respect to food (e.g., venetoclax [60]) and the gut microbiota that is altered by IF [20,74].

Overall, the currently available studies investigating fasting therapy on cancer patients are limited, with small sample sizes and limited real-world evidence. It is, therefore, challenging to recommend IF for CLL patients on novel therapies. Furthermore, if practicing any type of IF is desired by patients while on cancer therapy, it should be with caution and needs to be more firmly established. This review has highlighted the available data on the risks of TLS and GIB in CLL patients receiving FLNAs as monotherapies. It has also shed light on the pharmacological data that may provide some guidance points on the permissibility and risks associated with different IF patterns in this patient population. Finally, it has illustrated the need to fill many gaps in the literature, starting with clinical real-world data on the effects of IF on the outcomes of therapy with monotherapy novel agents in CLL as well as in combination with monoclonal antibodies. Future studies are also needed to identify risk factors and protective measures to individualize the decision of IF.

## 10. Conclusions

The allure of IF to cancer patients stems from the pre-clinical evidence suggesting that it improves the response to anticancer therapy and mitigates the associated side effects. This narrative review is the first of its kind to explore the possible effects of IF on the risks of TLS and GIB in patients with CLL. TLS and GIB are particularly concerning in CLL in the era of frontline venetoclax and BTK inhibitors. In fluid-restricted IF, patients are at a higher risk of dehydration, which may pose an additional contributing factor for developing TLS, particularly with venetoclax therapy. In contrast, during fasting conditions (fluid-restricted or fluid-liberal), the administration of venetoclax can adversely affect its absorption. Moreover, the higher levels of gastric acid during fasting conditions may exacerbate the risk of GIB in patients receiving BTK inhibitors.

The current evidence available on IF and FLNAs in CLL is insufficient to recommend IF in this population. Hence, the results here should not be relied upon as evidence to initiate IF for CLL patients treated with FLNAs. However, given the value that some patients may place on practicing IF for religious, nutritional, or other reasons, it is crucial to have a thorough discussion with patients about what is currently known about the risks and benefits of IF before starting any pharmacotherapy. In CLL, the treatment paradigm is shifting toward therapy personalization based on multiple factors including comorbidities, response to therapy, and patient preference. This review emphasizes the importance of an individualized risk assessment for TLS and GIB before allowing CLL patients to practice IF, particularly when they are receiving BTK inhibitors or venetoclax. All in all, it is vital to adopt a collaborative decision-making approach with patients to ensure that the treatment plan is safe, optimal, and in alignment with the goals of therapy as well as the patient’s values and expectations from treatment. Further clinical research is encouraged to safely deduce the permissibility of IF in CLL patients on novel agents.

## Figures and Tables

**Table 1:** Examples of some types of fasting practices.

Fluid-Liberal IF *: Fasting Practices That Allow Liquid Intake	Fluid-Restricted IF *: Fasting Practices That Prohibit Liquid Intake
***Baguan zhai (Buddhism; religious fasting)*** Practitioners abstain from meat and fish during fasting hours (from midday to the next morning on specific days).	***Ramadan (Islam; religious fasting)*** Practitioners abstain from all food and fluids (oral or intravenous) during fasting hours (daylight hours for 29 to 30 consecutive days).
***Lent (Christianity; religious fasting)*** Practitioners abstain from dairy products, meat, alcohol, eggs, and oil during fasting hours (daytime hours for 40 days).	***Proşadhopavāsa (Jainism; religious fasting)*** Practitioners abstain from satiating food, water, and desserts during days 8 and 14 of the lunar cycle.
***Calorie restriction for specific days of the week (non-religious fasting)*** Practitioners restrict calories for a specific number of days of the week (e.g., 5:2, where calorie restriction is performed on 2 days of the week, without specific dietary restriction). Liquids are allowed on fasting days.	
***Time-restricted eating (non-religious fasting)*** Practitioners abstain from any food during fasting hours (e.g., 16:8 is fasting for 16 h and allowing food consumption for 8 h). Water and non-caloric drinks (e.g., coffee) are allowed during fasting hours.	***Yom Kippur (Judaism; religious fasting)*** Practitioners abstain from food and fluids for a full 24 h for 1 day.
***Eat-stop-eat approach (non-religious fasting)*** Practitioners abstain from any food for 24 h on alternate days with 150% energy intake on non-fasting days.	

* This is not a comprehensive list of all types of religious and non-religious fasting practices. These are the IF practices described by Mandal et al. [27] and Templeman et al. [26].

**Table 2:** Ramp-up schedule of venetoclax monotherapy in CLL.

Week/Dose	Week 1	Week 2	Week 3	Week 4	Week 5 → Onward
400 mg/day					
200 mg/day					
100 mg/day					
50 mg/day					
20 mg/day					

**Table 3:** Incidence of TLS extracted from landmark head-to-head clinical trials of novel agents approved for CLL management.

Trial	ELEVATE-RR		ALPINE		CAPTIVATE
Comparison	Ibrutinib (*n* = 263)	Acalabrutinib (*n* = 266)	Ibrutinib (*n* = 324)	Zanubrutinib (*n* = 324)	Ibrutinib + Venetoclax (*n* = 159)
Any grade, *n* (%)	1 (0.4)	1 (0.4)	0	1 (0.3)	0 *
Grade ≥ 3–*n* (%)	1 (0.4)	1 (0.4)	0	1 (0.3)	0 *

* Percentage was reported among the adverse events that were identified in ≥2% of the included patients. For TLS, it was reported as 0% and the exact number is unavailable.

**Table 4:** TLS cases reported in FAERS among CLL patients treated with novel agent monotherapies.

Novel Agent	Ibrutinib	Acalabrutinib	Zanubrutinib	Venetoclax
Total number of all adverse events reported by June 2023	63,316	3694	760	35,886
Cases of TLS reported as monotherapies in CLL				
Total number of cases	73	36	3	195
Total number of serious cases	71	36	3	192
Deaths	20	2	0	68

**Table 5:** Incidence of hemorrhage extracted from landmark head-to-head clinical trials of novel agents approved for CLL management.

Trial	ELEVATE-RR		ALPINE		CAPTIVATE
Comparison	Ibrutinib (*n* = 263)	Acalabrutinib (*n* = 266)	Ibrutinib (*n* = 324)	Zanubrutinib (*n* = 324)	Ibrutinib + Venetoclax (*n* = 159)
Hemorrhage					
Any grade, *n* (%)	135 (51.3)	101 (38)	134 (41.4)	137 (42.3)	Not reported
Grade ≥ 3–*n* (%)	12 (4.6)	10 (3.8)	12 (3.7)	11 (3.4)	
Major hemorrhage					
Any grade, *n* (%)	14 (5.3)	12 (4.5)	14 (4.3)	12 (3.7)	3 (2)
Grade ≥ 3–*n* (%)	12 (4.6)	10 (3.8)	12 (3.7)	11 (3.4)	2 (1)

**Table 6:** GIB cases reported in FAERS.

Novel Agent	Ibrutinib	Acalabrutinib	Zanubrutinib	Venetoclax
Total number of all adverse events reported by June 2023	63,316	3694	760	35,886
Cases of GIB reported as monotherapies in CLL				
Total number of cases, *n* (concomitant antiplatelet or anticoagulant)	191 (207)	5 (NA)	1 (NA)	19 (NA)
Total number of serious cases, *n* (concomitant antiplatelet or anticoagulant)	189 (205)	5 (NA)	1 (NA)	19 (NA)
Deaths, *n* (concomitant antiplatelet or anticoagulant)	26 (29)	0	0	10 (NA)

NA: not available.

**Table 7:** Literature- and drug-label-based comparative food interactions of BTK inhibitors approved for CLL food management.

BTK Inhibitor	Ibrutinib	Acalabrutinib		Zanubrutinib
PK Data	Food increased AUC by 2-fold Food increased Cmax by 2- to 4-fold	Capsules: For high-fat and high-caloric meals: No effect on mean AUC compared to fasting conditions Decreased Cmax by 73% Delayed Tmax by 1–2 h	Tablets: For high-fat and high-caloric meals: No effect on mean AUC compared to fasting conditions Decreased C_max_ by 54% Delayed T_max_ by 1–2 h	No significant clinical effect
Drug Label	Can be given without regard to food *	Capsules and tablets: Can be given without regard to food		Can be given without regard to food

PK: Pharmacokinetic. * Label recommendation assumes that repeated fasting condition is unlikely.
